# Patient’s perceptions of presbycusis and associated tinnitus counselling practices within the KwaZulu-Natal province

**DOI:** 10.4102/sajcd.v69i1.868

**Published:** 2022-09-07

**Authors:** Kerusha Bhojraj, Vuyelwa Z. Peter

**Affiliations:** 1 Department of Health, Durban, South Africa; 2 Discipline of Audiology, University of KwaZulu-Natal, Durban, South Africa

**Keywords:** patient outcomes, recommendations, patient-centred care, presbycusis, tinnitus

## Abstract

**Background:**

Counselling manages the psychosocial effects of presbycusis and associated tinnitus, which is best conducted through patient-centred care (PCC). However, there is a paucity of research on implementing PCC within audiology and on patients’ perceptions of counselling practice, making the focus on patient benefit and satisfaction crucial. Furthermore, PCC has been documented to be suitable in addressing the psychosocial effects of hearing loss and associated tinnitus, as it focuses on the adult patient playing an integral part of the management processes, providing improved patient outcomes.

**Objectives:**

This study aimed to explore patients’ perceptions of presbycusis and associated tinnitus counselling practices by audiologists within KwaZulu-Natal (KZN).

**Method:**

Qualitative phenomenological semi-structured telephonic interviews were conducted through purposive sampling, consisting of seven patients with presbycusis and associated tinnitus who were recruited from private and public facilities in KZN. Data were analysed through hybrid thematic analysis following Braun and Clark’s steps.

**Results:**

Six categories were identified: perceptions on counselling methods, efficacy and tools, audiological rehabilitative training, multicultural sensitivity, patient satisfaction and recommendations on improving counselling practices as PCC adaptation. Themes were then extracted from these categories. The overall outcome of the study found that patients had positive perceptions of methods and tools, and audiologists were viewed as adequately trained. Furthermore, they were satisfied and benefitted from counselling practices and found clinicians to be culturally sensitive in their practice. However, recommendations were made towards linguistic sensitivity and satisfaction evaluations.

**Conclusion:**

Therefore, implementing PCC into counselling practice may achieve positive patient perceptions, thus highlighting the need to identify barriers and improve the implementation of PCC into practice, especially in resource-constrained contexts.

## Introduction

Presbycusis is an age-related hearing loss, known as the most common cause of hearing loss in ageing adults (Sogebi, Olusoga-Peters, & Oluwapelumi, 2014). Research has determined that presbycusis can start to manifest in adults as early as 30 years of age (Kim & Yeo, 2015). It directly and negatively impacts physical, cognitive, social and emotional functioning of patients, leading to lowered self-esteem, depression and social isolation (Lee, 2013; Sprinzl & Riechelmann, 2010). Apart from these psychosocial effects of presbycusis, a common side-effect is tinnitus, a noise in the ear that is described by patients as a ringing or buzzing sound (Cunningham & Tucci, 2017; Lee, 2013; Zhang, Yu, & Ruan, 2020). The effects of tinnitus are similar to presbycusis, ultimately leading to deterioration in quality of life (Cunningham & Tucci, 2017; Ferreira, Ramos, & Mendes, 2009; Sogebi et al., 2014).

When zeroing in on presbycusis and tinnitus within the South African context, data is scarce. However, some research in public healthcare facilities revealed a hearing loss prevalence of 17.5%, with presbycusis being the predominating cause of hearing loss (Louw, Swanepoel, Eikelboom, & Hugo, 2018), causing a possibly significant prevalence of presbycusis in South Africa (SA). Furthermore, the World Health Organization (WHO) (2018) indicated that 4.57% of the population in sub-Saharan Africa (SSA) is affected by a disabling hearing loss, which is considered to be a loss of hearing greater than 35 decibels in the better hearing ear (WHO, 2018). Furthermore, the WHO (2018) predicted a rapid increase in prevalence in the years to follow. Sub-Saharan Africa and SA prevalence on hearing loss and the predicted increase (Louw et al., 2018; WHO, 2018) indicates the necessity of exploring presbycusis and associated tinnitus management to better understand the impact on the population afflicted, thus allowing for improved and beneficial management strategies to be implemented.

Apart from SSA, presbycusis and associated tinnitus affect millions of adults worldwide (Kidd & Bao, 2012). A study conducted in India on 2695 adult patients in the age range of 60 and above revealed presbycusis as the predominant cause of hearing loss and a tinnitus prevalence of 16.81% (Thirunavukkarasu & Geetha, 2013). Similar research conducted in Japan on 14 423 adults between the ages of 49 and 79 presenting with hearing loss (predominantly presbycusis) revealed an 11.9% prevalence of tinnitus (Fujii et al., 2011). Therefore, these studies demonstrate a common trend of a coexisting relationship between presbycusis and associated tinnitus and an increase of prevalence with a corresponding increase of age (Fujii et al., 2011; Thirunavukkarasu & Geetha, 2013). These reported studies are from Asia and Pacific, with a paucity of similar studies from SSA reflecting associations between presbycusis and tinnitus. This creates a gap in knowledge and further strengthens the need for exploration of presbycusis and associated tinnitus, in order to better understand the impact on the population afflicted for improved management strategies and to close knowledge gaps.

Current and most common management strategies for presbycusis and associated tinnitus include compensating for the hearing loss through the use of hearing assistive devices (Swain, Nayak, Ravan, & Sahu, 2016). Whilst advances in technology have led to better management options for presbycusis and associated tinnitus, there is no cure (Swain et al., 2016). This intensifies the need for further exploration on the management of presbycusis and associated tinnitus. Over decades of research, it has been indicated that compensation through amplification alone does not adequately address the negative impacts of presbycusis and associated tinnitus on quality of life, as mentioned earlier. Consequently, advantageous management of presbycusis and associated tinnitus goes beyond compensation to include counselling (Boothroyd, 2007; Chisolm & Arnold, 2012; Hawkins, 2005; Parham, Lin, Coelho, Sataloff, & Gates, 2013).

Counselling is conducted post-assessment right up until discharge of the patient, which serves as a means of imparting knowledge to patients and assisting them in adjusting to their hearing loss and its impact on quality of life (American Speech-Hearing Association [ASHA], 2012; British Society of Audiology [BSA], 2016). Whilst counselling may be deemed a crucial part of management for presbycusis and associated tinnitus, there is a lack of research on the benefit of counselling practices within South Africa. It has further been anecdotally discovered that counselling practices within South Africa require exploration regarding appropriateness and patient outcomes.

Research in South Africa has revealed unpleasant patient experiences during healthcare delivery, resulting from communication breakdowns and lack of respect for patient preferences, autonomy and socio-economical discriminatory experiences from healthcare providers (De Man et al., 2016; Peltzer, 2009). Although research on patient outcomes within the field of audiology in South Africa is limited, with the known undesirable patient experiences from generalised healthcare delivery, providing counselling practices beneficial to patients within the patient-centred care (PCC) approach is crucial. Patient-centred care is a framework that proposes that the patient’s individual needs should be the point of focus when providing services and promoting patient satisfaction (Nkrumah & Abekah-Nkrumah, 2019). Over the years, PCC has been advocated in healthcare delivery and intends to improve patient outcomes by offering services that are both meaningful and valuable to the individual patient (Epstein & Street, 2011; Nkrumah & Abekah-Nkrumah, 2019; Rathert, Wyrwich, & Boren, 2013). Therefore, the effectiveness of PCC in practice may be represented by patient satisfaction by focusing on the patient’s perceptions (Smith & Dunham-Taylor, 2018). Substantiated by Sladdin, Chaboyer, and Ball (2018), who conducted a study exploring 11 patients’ perceptions of PCC by focusing on their experiences from dietetic services, it was found that adopting PCC into practice improved patient satisfaction. Based on suggestions by Sladdin et al. (2018), implementing PCC during counselling practices may be the favoured approach in improving patient satisfaction. This suggestion can further be considered for presbycusis and associated tinnitus in the SA context, as there is already limited documented evidence of PCC application. However, research has indicated that in SSA, PCC was found to be poorly implemented in healthcare because of strained resources, poor interpersonal interactions between patients and healthcare providers and a lack of focus on training healthcare providers regarding psychosocial dimensions of illnesses (De Man et al., 2016). This is no exception in South Africa, with research indicating that patients experience poor quality of care (Maphumulo & Bhengu, 2019). More specifically, research suggests that audiologists in South Africa reported that time constraints, language barriers, lack of training and limited resources have negatively impacted their service provision (Makhoba & Joseph, 2016). These are contributing factors that hinder PCC implementation amongst audiologists in South Africa, leading to dissatisfied patient outcomes. There is still a paucity of research on PCC implementation within the field of audiology, with literature accounting for audiologists’ first-hand descriptions of patient outcomes and satisfaction from presbycusis and associated tinnitus counselling. Meibos, Muñoz and Twohig (2019) suggested that to improve counselling practice or skills, audiologists should better understand patients. Therefore, exploring patients’ perceptions on presbycusis and associated tinnitus counselling practices may improve patient outcomes.

DiLollo and Neimeyer (2020) described counselling practice in audiology in terms of components of a clinician–client framework, a term used interchangeably with PCC. The audiologist’s main focus is on the individual needs of the patient and the patient’s significant others in order to facilitate patient participation, assisting patients to adjust and cope with the psychosocial impacts of hearing loss (DiLollo & Neimeyer, 2020). Consequently, PCC serves as a fundamental influence in beneficial counselling practices amongst audiologists. However, there is a gap in literature on the nature and outcomes of PCC in audiologists’ counselling practices and a need to conceptualise PCC within audiology (Grenness, Hickson, Laplante-Lévesque, & Davidson, 2014). Therefore, focusing on patients’ perceptions and conceptualising PCC is crucial in closing these research gaps.

To conceptualise PCC into counselling practices, components of PCC need to be focused upon. Patient-centred care components are based on the eight picker principles: respect for the patient’s values, preferences and expressed needs; coordination and integration of care; information sharing; comfort and support to the patient and significant others; involvement of significant others; continuity; and access to care (Picker Institute Europe, 2020).

**FIGURE 1:**
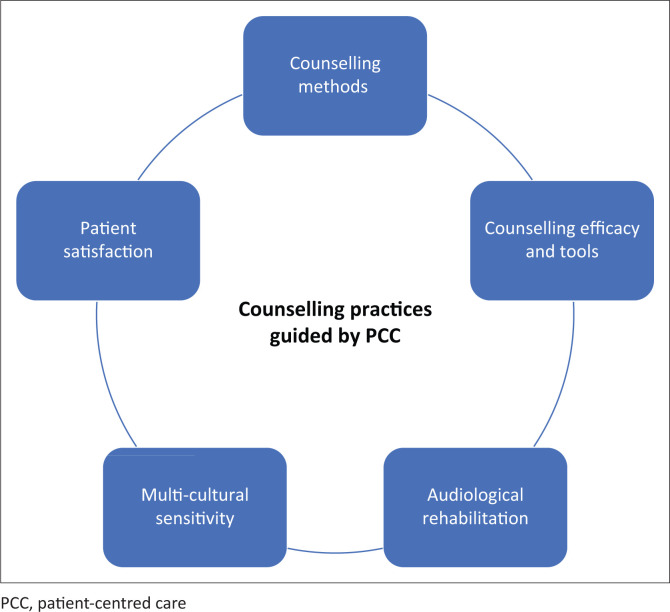
Conceptual framework adapted from patient-centred care, depicting aspects of counselling practices guided by patient-centred care that have been adopted for this study.

Therefore, this study conceptualised a framework as depicted in Figure 1, focusing on components of PCC in counselling practices. The framework is described in various aspects excerpted as:

- counselling methods: the forms or procedures used by audiologists during counselling practices, which include informational or adjustment counselling (ASHA, 2012)
- counselling efficacy and tools: the benefits or helpfulness of counselling practices measured through measurement tools (BSA, 2016)
- audiological rehabilitative training: the audiologist’s training, skills and exposure to counselling practices
- multi-cultural sensitivity: diverse cultural and linguistic backgrounds of patients that should be considered whilst delivering services
- patient satisfaction: the patient’s overall benefit and satisfaction from counselling practices

A scoping review of 18 articles found that audiologists have limited knowledge of patients’ perceptions of counselling practices because of the paucity of research and require better knowledge and training for improved counselling practice (Meibos et al., 2017). Furthermore, PCC may be poorly implemented in SSA, despite review literature asserting increased patient satisfaction with its implementation (De Man et al., 2016). There is a gap in research regarding the above-discussed aspects of PCC being implemented in counselling sessions of patients with presbycusis and associated tinnitus. This gap may have a direct impact on the patient’s perception of the service they have received. Furthermore, research on patients’ perceptions of counselling practices specific to the South African context is scarce. Thus, this study would explore patients’ perceptions of audiologists’ practices of presbycusis and associated tinnitus counselling, in order to improve on practices within a PCC framework.

## Methodology

### Aims

This study aims to explore patients’ perceptions of presbycusis and associated tinnitus counselling practices offered by audiologists, within KwaZulu-Natal (KZN).

### Study design and context

The study followed a qualitative, phenomenological design concerned with the participants’ lived experience, using semi-structured telephonic interviews developed by the researcher to describe patients’ perceptions of counselling practices. Furthermore, the phenomenological design allowed for in-depth perspectives of patients receiving presbycusis and tinnitus counselling by exploring their experiences (Brink, Van der Walt, & Van Rensburg, 2012). The phenomenological design allowed for in-depth perspectives of the patients receiving presbycusis and associated tinnitus counselling by exploring their experience through information (Brink et al., 2012). Telephonic interviews provide an effective and economical method of gathering data for a wide geographic sampling population (Fox, Hunn, & Mathers, 2009), making it a suitable design for this study. In addition, the study was conducted during the COVID-19 pandemic; therefore, telephonic interviews reduced the risk and spread of infections.

### Study site, population and sampling technique

Participants were sourced from both public and private sectors within four districts of KZN. These districts were a mix of rural and urban, with urban districts being eThekwini and uMgungundlovu and rural being Zululand and King Cetshwayo. Languages predominant in KZN are English and isiZulu, with other African languages also spoken within this province (Pillay, Tiwari, Kathard, & Chikte, 2020). However, patients are seen by audiologists in KZN that are predominantly English-speaking (Pillay et al., 2020). Seven participants were interviewed. Of these seven participants, one was isiZulu-speaking and seen by an isiZulu-speaking clinician. This participant had a good knowledge of English; however, a trained isiZulu interpreter was sought throughout the data collection process. The other six participants were English-speaking and were seen by clinicians speaking English. Furthermore, all participants were literate. The sample size of seven participants was decided upon after consulting a statistician, which allowed for a sample large enough to gain insight into the phenomenon yet small enough to gain in-depth data (Vasileiou, Barnett, Thorpe, & Young, 2018). The participants had to meet the inclusion criteria that required them to be adults within KZN, diagnosed with tinnitus and presbycusis, with severity between mild to profound. Participant demographics are represented in Table 1.

**TABLE 1:** Participant demographics.

Participant	Age	Gender	Race	Public or Private	Urban or Rural
1	67	Male	White	Public	Urban
2	45	Female	Indian	Public	Urban
3	55	Female	White	Private	Rural
4	57	Female	Indian	Public	Rural
5	69	Female	Black	Public	Rural
6	34	Female	Indian	Public	Urban
7	45	Female	Indian	Public	Urban

Furthermore, purposive sampling was used to ensure that participants were representative of the phenomena-based research (Brink et al., 2012).

### Data collection tool

Semi-structured telephonic interviews were conducted to minimise contact with patients because of the COVID-19 pandemic that restricted physical contact with participants. Therefore, telephonic interviews allowed for an in-depth conversation whilst reducing chances of COVID-19 transmission. Furthermore, telephonic interviews were conducted with patients using open-ended questions that were guided by the structure and nature of the Hearing Handicap Inventory for the Elderly (HHIE) and the WHO Disability Assessment Schedule (WHODAS) questionnaires, in order to derive information on self-care, participation, interpersonal and life activities (BSA, 2016). The interview was focused on counselling based on interaction between audiologist and patient from the post-assessment stage until the point of discharge. The interview schedule was guided by the Picker Patient Experience Questionnaire, which explores patients’ experiences with hearing loss (Jenkinson, Coulter, & Bruster, 2002). The Picker principles provide a guideline for PCC and are included in the PCC framework utilised for this study. The semi-structured interview schedule included a biographical section to obtain patient information, followed by sections to obtain patients’ perceptions of counselling practices: these included counselling methods, counselling efficacy and tools, audiological rehabilitative training of audiologists, multicultural sensitivity of audiologists and patient satisfaction. The interview schedule ended with a section focusing on patients’ recommendations on improving counselling practices. Furthermore, Al-Abri and Al-Balushi (2014) described patient satisfaction as the patient’s emotions, feelings and perceptions of health services rendered, whilst other researchers (Ahmad, Nawaz, Khan, Khan, & Rashid, 2015) described patient satisfaction as the relationship between the patient’s expectations of health services and their perceptions of services received. Therefore, the interview schedule was developed to obtain information to cover both these descriptions of patient satisfaction.

### Data collection procedure

Gatekeeper permission was obtained from KZN Department of Health (DOH) and clinicians in private practice. Private and public audiologists practising in KZN were contacted to obtain details of patients who met the stipulated criteria, after receiving patients’ consent to participate in the study. Information and consent forms were sent to patients through their respective audiologists and then shared directly with the patient via a document link after they agreed to participate. Once patients agreed to participate, the date and time were set to conduct the telephonic interviews that focused on counselling, based on interaction between audiologist and patient from the post-assessment stage until the point of discharge. The interview was audio-recorded and verbatim transcription was carried out.

### Data analysis

This study used a hybrid approach of thematic analysis of data that includes both inductive and deductive measures (Swain, 2018). The hybrid thematic analysis allowed the researcher to obtain rich data on patients’ perceptions of counselling practices whilst focusing on the theoretical framework providing for an analysis of the phenomenon researched (Swain, 2018). This approach allowed for the analysis for identifying, analysing and reporting themes from the data collected using NVivo12 software. Transcription of audio recordings of seven participants was conducted to arrive at a written document useful for qualitative data analysis (Swain, 2018). The transcriptions were imported onto NVivo12 software for coding, analysis, management and representation of themes. Thematic analysis was conducted to derive insight into the phenomena studied (Swain, 2018).

### Rigour and Trustworthiness

Rigour was assessed using a four-dimensional criterion: building of a research team, data collection preparations, participation in the research and consistency of the study (Forero et al., 2018). This was performed by ensuring that the researcher completed an ethics course, adapting data collection tools from the existing literature where reliability and validity have already been established (Jenkinson et al., 2002; Picker Institute Europe, 2020), consulting a statistician during planning phases of participants involved in this study and maintaining consistency throughout the study by following ethics discussed later (Forero et al., 2018). Therefore, rigour was ensured through the four-dimensional criterion that assisted the researcher in reaching objectives through informed data analysis whilst being sensitive towards interpretation bias. Rigour was further demonstrated by developing a code manual, summarising data, identifying initial themes, applying template of codes, connecting codes, identifying themes, corroborating and legitimating coded themes (Fereday, 2006). Rigour was further ensured by developing the data collection tools with guidance from already existing literature where reliability and validity have already been established (Picker Institute Europe, 2020) and conducting a pilot study prior to the main study. Furthermore, the pilot study ensured adequacy, ordering and comprehensiveness of the research tools used (Regmi, Waithaka, Paudyal, Simkhada, & Van Teijlingen, 2017). Trustworthiness was maintained throughout the study through transparency and by ensuring that participants fully understood and consented to the study before data collection (Fereday, 2006). To enhance the authenticity of data findings, a member check was conducted, all participants of the study were provided with the written transcript of their interview (Fereday, 2006) and all participants were further invited to approach the researcher if there were any disagreements on transcriptions and interpretations.

### Ethical considerations

Ethical clearance was obtained from the Humanities and Social Sciences Ethics Committee of the University of KwaZulu-Natal (UKZN) with reference number HSSREC/00002114/2020. The study adhered to the Declaration of Helsinki code of conduct for ethical research (Goodyear, Krleza-Jeric, & Lemmens, 2007).

## Results and discussion

The study focused on a framework based on the fundamental components of PCC during counselling sessions offered by the audiologist, as discussed earlier in the article. Aspects of counselling practices framed the study. Six main categories were then identified during analysis: perceptions on counselling methods, efficacy and tools, audiological rehabilitative training, multicultural sensitivity, patient satisfaction and recommendations on improving counselling practices. These themes are depicted in the given developed concept map.

**FIGURE 2:**
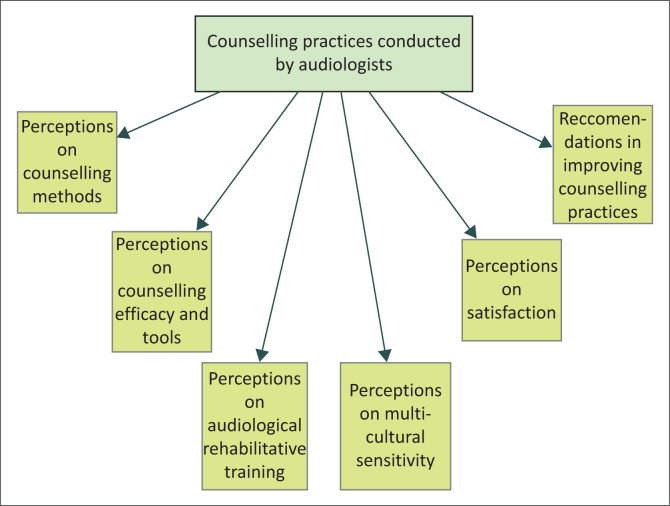
Concept map depicting themes developed within this study.

The concept map given in Figure 2 identifies the categories generated from the study, closely linked to the study’s conceptual framework. An overview of results from the study is depicted in Table 2.

**TABLE 2:** Representation of categories and themes found in the study.

Categories	Themes
1. Perceptions on counselling methods	Informational counselling Adjustment counselling Group counselling Family counselling
2. Perceptions on counselling efficacy and tools	Tools/resources used during counselling
3. Perceptions on audiological rehabilitative training	Capability/incapability of audiologists Shared decision making
4. Perceptions on multicultural sensitivity	Satisfaction and benefit from multicultural sensitivity
5. Perceptions on patient satisfaction	Benefit and satisfaction
6. Recommendations made by participants to improving counselling practices	Improving counselling practices

### Category 1: Perceptions on counselling methods

English and Archbold (2014) described informational counselling as a form of teaching in which the audiologist imparts knowledge to the patient and significant others related to the hearing diagnosis. All participants (*n =* 7) reported an overall positive perception of counselling methods used by audiologist to manage presbycusis and associated tinnitus. These participants had positive regard for audiologists who addressed their need for information and provided them with skills to cope with their problem. All participants reported that they were provided with information on their hearing impairment and assistive device. However, despite this overall positive perception of audiologists meeting their need for information on presbycusis and associated tinnitus, all patients struggled to confidently explain the type of hearing loss and assistive devices that they used. It is speculated that this may be attributed to some insufficiencies of providing information during informational counselling in terms of information that is exchanged or the manner in which information is exchanged. Research has revealed that insufficiencies during informational counselling can infringe upon a patient’s decision-making (Shezi & Joseph, 2021). Therefore, audiologists need to utilise counselling strategies to ensure that information provided is understood and internalised for patient’s informed decision-making (Shezi & Joseph, 2021). Apart from informational counselling, the BSA (2016) described adjustment counselling as exploring the patient’s experiences and identifying the conflicts and emotions they go through to deal with the diagnosis. The most common adjustment or lifestyle change experienced by a majority (6) of participants was based on the use and maintenance of the hearing device:

‘The only lifestyle changes would be to take care of the equipment, the cleansing part of it, the looking after it, how to deal, how to manage it. Working with the battery and how long the batteries last. That aspect of giving attention to the hearing aid is somewhat of a small bracket of lifestyle changes.’ (Participant 1, 67, Male)

Apart from this, two out of seven participants reported communication strategies and repairs being discussed in their adjustment counselling session. Therefore, five out of seven participants did not report on lifestyle changes to accommodate or adjust to their life with tinnitus. Therefore, these were interpreted as undesirable responses, as the primary need for a majority of participants (five), adjusting to presbycusis and associated tinnitus with lifestyle changes required to deal with the disabling hearing loss, was not fulfilled for the patient:

‘If I can’t hear when the person speaks then I must switch off everything and turn my full attention to the person speaking to me, also she told me if I’m speaking to someone that I should tell them to speak slowly and clearly so that I may understand them.’ (Participant 2, 45, Female)

The narrative from Participants 1 and 2 demonstrates that whilst audiologists are providing some level of adjustment information to the assistive device counselling, the patient’s perceived benefits are based mainly on adjusting their lives to accommodate the hearing device. Whilst this may provide for adjustment to an extent, it is not adhering fully to patients’ experiences or adjustments to the psychosocial impacts of presbycusis, which go beyond just compensating for a hearing loss (Parham et al., 2013). Similar studies in medicine revealed patients’ experiences of inefficiency from adjustment counselling because of follow-up dialogue, rushed time or lack of follow-up sessions, which this study suggests being similar practice within KZN (Walseth, Abildsnes, & Schei, 2011). These insufficiencies have been noted in previous audiological research conducted in South Africa, revealing that audiologists’ inefficiency of counselling was because of audiologist bias, lack of information provided to patients and turning a deaf ear to the individual needs of the patient (Shezi & Joseph, 2021). Shezi and Joseph’s (2021) findings on the inefficiency of counselling are corroborated in this study. This study asserts that audiologists therefore need to include improved information exchange during counselling to ensure that patients can confidently apply and participate in informed decision-making during the rehabilitation process (Shezi & Joseph, 2021). This will improve the perceived benefit of counselling offered by audiologists to patients. The similarities in inefficiency from methods of counselling experienced by participants may be improved by adopting PCC into practice, which will provide information-sharing, educate patients, ensure respect of patients’ preferences and emotional support and include patients’ significant others (Picker Institute Europe, 2020).

Further methods of counselling are the delivery through individual or group sessions (BSA, 2016). None of the participants interviewed reported participating in group counselling, and all participants described a preference for individual or family counselling over group counselling. Furthermore, one out of seven participants reported that they were advised by the audiologist to attend group counselling. The remaining six participants were not offered the option of group counselling. However, despite the limited offering of group counselling sessions, none of the participants showed preference for group counselling over individual or family counselling, as seen by the narrative of Participants 2 and 3. This was similar to the initial study, with audiologists stating an inclination towards individual sessions and lacking group counselling.

Participant 2 (45, Female) stated, ‘I preferred one on one it was more private’. Participant 3 (55, Female) said, ‘It was individual, yes [*preferred it that way*], because it’s one on one and she [*the audiologist*] could answer my questions’.

Research conducted in Australia suggests that the most commonly used approach for conducting counselling is through group sessions (Ekberg, Meyer, Scarinci, Grenness, & Hickson, 2015), as it allows for sharing of experiences, addressing coping strategies and counselling more than a single individual in one sitting. Contrary to that study, however, it was found in this study that none of the participants interviewed reported being part of group counselling or showed a preference for group counselling. This study perceives it as placing them at a disadvantage, as they are missing out on the many documented benefits of group counselling. Furthermore, most participants interviewed were not offered the option of group counselling; it could be possibly attributed to the COVID-19 pandemic causing disruptions to traditional service delivery, as measures to curb the spread of the disease required minimising contact (Pfattheicher, Nockur, Böhm, Sassenrath, & Petersen, 2020). Despite the COVID-19 pandemic, anecdotally it does not appear that audiologists are providing options for group counselling for patients as part of counselling practice in KZN. This study advocates that those audiologists should consider offering group counselling sessions whilst adhering to safety protocols, allowing patients the opportunity to opt out, in line with the PCC principle of offering a variety of counselling methods. Furthermore, this preference of individual or family counselling over group counselling was attributed to patient privacy concerns and fear of embarrassment or judgement, as depicted in the following narrative:

‘Well, I don’t suppose you ever want everybody else to know, because people are always inquisitive and curious to know your business, and they’re just sitting there not minding their own business.’ (Participant 1, 67, Male)

Participant 1 (67, Male) showed preference towards individual or family counselling, as did other participants as seen by the narrative of Participant 4 (57, Female), who said, ‘I can manage on my own. I’m a very private person. I don’t like discussing my private life’.

Participants’ negative perception towards group counselling may be influenced by insufficiencies of information given to the patient from audiologists, impacting their informed decision-making (Shezi & Joseph, 2021) and causing patients to opt out of group counselling. Therefore, a link is created with the patient’s inclination to individual or family counselling and informational counselling. This link is suggestive that a lack of information may contribute to a patient’s negative perceptions of group counselling. These negative perceptions by patients may be influenced by providing patients with additional information on the benefits and impacts of group counselling whilst assuring patients of their privacy. This article recommends further investigation in terms of research to benchmark group counselling practices specific to the South African context that may improve patient outcomes as depicted in literature (Ekberg et al., 2015).

Apart from group sessions, most participants reported a lack of family or significant others being present during counselling and reported negative perceptions on the lack of involvement of significant others during counselling. Ekberg et al. (2015) surveyed the involvement of family during the audiological rehabilitation process that included audiologists, patients and patients’ families. This study reported that families present at sessions were not invited into conversations between patient and audiologist; only 12% had family participation, despite family members’ willingness to actively participate and show support. Despite literature by Ekberg et al. (2015) and patients who prefer family or significant others’ involvement during counselling, it is not fully offered to patients within KZN by audiologists, as seen in the following narrative:

‘It makes a great impact because I feel if they [*family or significant others*] are there and if something happens, they can guide me if I don’t hear anything. Makes me feel safer.’ (Participant 7, 45, Female)

This lack of involvement from family or significant others of patients may impact PCC dimensions and patient benefits because PCC ideals advocate for patients’ active participation and involvement of significant others (Picker Institute Europe, 2020). Ekberg et al. (2015), further revealed that audiologists do not have a full appreciation of how to incorporate and successfully use significant others in the counselling session for patient benefit. This study shares similar sentiments with these authors, because the lack of inclusion and appropriate use of significant others in counselling sessions has been seen in its findings. This needs to be considered in practice as most participants (*n* = 4) showed an inclination towards family counselling over group counselling, as they reported feeling supported emotionally, safe to express themselves and certain that information was not missed or misunderstood, as explained by Participant 7 in the given narrative. However, none of the four participants were given the option to bring a family member for future sessions. Lack of an option for family or significant others’ involvement may also impact counselling efficacy. Therefore, audiologists are urged to encourage inclusion and invite the significant other to the sessions (Ekberg et al., 2015). Furthermore, for effective carry-over of counselling strategies shared with the patient, the significant other can be a facilitator in the patient’s natural environment (Ekberg et al., 2015). For further effective carry-over to be facilitated, appropriate tools such as measurement scales are required during the counselling session to improve the value of intervention.

### Category 2: Perceptions on counselling efficacy and tools

For the purposes of this study, efficacy of counselling refers to the benefits or helpfulness of counselling practices measured by tools such as checklists or questionnaires (BSA, 2016). To assess the efficacy of presbycusis and associated tinnitus counselling practices, audiologists must ensure the use of appropriate measurement tools, with the most common in use for adults being the HHIE, the Tinnitus Handicap Inventory and the WHODAS II (BSA, 2016). All seven participants demonstrated positive inclination towards the use of tools to rate the efficacy of the counselling practice. However, despite the positive perception, all participants mainly commented on audiology booths as a tool used by the audiologists, as can be seen in the given narrative. None of the participants reported use of questionnaires or checklists during the session to assess effectiveness of counselling, as is also portrayed in the given the following narrative:

‘They used a silent room and they had headphones and stuff that they put on different ways and then there was sounds that they registered on a machine computer and then programmed it, they used charts and then she actually had the physical stuff here. They had the hearing aid and the batteries and then demonstrated how it needed to be used.’ (Participant 1, 67, Male)

It was found that participants described the equipment used during assessment of hearing as the tools or resources used during counselling practices. This misunderstanding by patients of tools or resources may be attributed to insufficiencies during the counselling process in informing patients of respective tools or resources used and their purpose during practice. This study identified desynchrony in counselling practice between audiologists and patient perceptions in terms of understanding the tools, their use and efficacy, which may be because of time constraints. However, such insufficiencies may impact patient satisfaction and benefit. This study speculates that audiologists in KZN do not routinely use checklists or questionnaires to measure the impact or validation of presbycusis and associated tinnitus on a patient’s quality of life. Whilst the reasons are unknown, it could be attributed to lack of access to tools or resources. The lack of using measurement scales may impact patient benefit from counselling, as there is no full appreciation of the psychosocial impact on quality of life because measures that foreground the severity that may be used to guide counselling practice are hardly used (Parham et al., 2013). Further impacting on and linking to adjustment counselling practices, since the psychosocial impacts of presbycusis and associated tinnitus are not fully undertsood and measured, therefore, patients may not be appropriately adjusting to the impacts of hearing loss and tinnitus. The use of these validation tools during counselling practice is imperative as it affects patients’ overall progress towards goals of improved quality of life and implements integrated care amongst audiologists (BSA, 2016), in line with PCC components.

Furthermore, all participants reported counselling to be effective based on the fitting of hearing devices, placing the patient at a disadvantage because of insufficient information or knowledge on what they expect to achieve from counselling, which goes beyond just compensating for a hearing loss but also includes dealing with the psychosocial impacts (Parham et al., 2013). Audiologists’ insufficiently effective use of validation tools may negatively impact the patient’s outcomes or satisfaction. A study conducted in South Africa by Makhoba and Joseph (2016) shared similar sentiments, as it found that audiologists’ perceived limitations during undergraduate training might contribute to a waning, inefficient use of tools in counselling practices (Makhoba & Joseph, 2016). This can be evident in counselling practice where validation tools are not used. In addition, accessibility of the tools is another concern, as they can be costly and may have linguistic limitations as they are manufactured in developed countries (Makhoba & Joseph, 2016). When probed further, participants described the audiologist’s efficacy during counselling to be attributed to an ultimate diagnosis of their hearing loss alone, which allowed for management options such as compensating for the loss but lacked in management of psychosocial impacts of presbycusis and associated tinnitus. This may be attributed to the patient’s limited understanding of counselling outcomes, which may be linked to insufficiencies during information exchange with audiologists (Shezi & Joseph, 2021). This may further be associated with audiologists’ undergraduate training and exposure, impacting their management practice.

### Category 3: Perceptions on audiological rehabilitative training

Audiologists have indicated that limitations in training negatively impact their counselling practices (Makhoba & Joseph, 2016). Therefore, training focused on counselling skills may have a positive impact on audiologist counselling practices. All (*n* = 7) participants in this category expressed their perceptions of audiologists to be well trained and capable of counselling, with the main attributes being kindness, understanding and patience, as is portrayed in the given narrative. Participant 1 said, ‘we were treated with the highest respect and honour and decency and kindness’. This study speculates the limited offering of adjustment counselling and use of validation tools can be attributed to similar training exposure.

The patients’ perceptions are based on their experiences with services rendered to end-users, which might be conflated with audiologists’ adequate training and exposure. The researcher was mindful and appreciated that the participants’ perceptions of audiologists in terms of training competence are different based on differing levels of knowledge of the participants. Therefore, for the purpose of this study, the study reported on how patients perceive audiologists’ competence. Furthermore, there is an appreciation that audiologists’ competence measurement criteria are different for the patients and audiologists. As supported by Epstein and Street (2011), who suggested that if audiologists show mindfulness and empathy whilst being informative to patients, their role in the patient–audiologist dynamic will change from audiologists being viewed as authority figures to forming a partnership with patients, bringing about collaboration and improved patient participation. Audiologists require a set of micro-skills that will allow them to show mindfulness and empathy (ASHA, 2012); such micro-skills, amongst others, include listening, empathy and responding to patients (Beck & Kulzer, 2018). These skills are used subjectively with patients and may be developed over time with practice and training (Beck & Kulzer, 2018). Furthermore, Beck and Kulzer (2018) suggested audiologists’ skills may impact shared decision-making between patient and clinician. Shared decision-making is critical within the PCC framework (Picker Institute Europe, 2020). This study found that the majority of participants (six), felt that they were a part of the decision-making process during counselling. The practice of including patients in the decision-making process for assistive devices is appreciated as it is supported by Makhoba and Joseph (2016), who found that if patients are part of the selection of the device then there are high chances of acceptance. However, one participant reported to have not been involved in the decision-making process and attributed this to being treated at a public facility, as portrayed in the given narrative. Participant 5 (69, Female) stated, ‘I wasn’t there when they were making the decisions for my hearing aid; this is a government hospital’.

Therefore, this study found that whilst a majority of participants reported having been included in shared decision-making, these were decisions solely on their hearing device, and there are different practices between private and public sectors based on shared decision-making.

Studies have suggested that audiologists demonstrate varied activities during counselling practises by utilising closed or open-ended questions, taking charge as authoritative figures during the counselling session or allowing the patient to lead the session (Pryce et al., 2018), depending on their training and skill level (Pryce et al., 2018). However, literature focused on this study advocates for PCC that allows patients to become active participants in shared decision-making (Picker Institute Europe, 2020). Therefore, Pryce et al. (2018) suggested that clinicians should better adapt their therapeutic skills to improve shared decision-making. (Picker Institute Europe, 2020). A participant from the public sector reported to have not been involved in the decision-making process, as can be seen in the narrative from Participant 5 depicted here, whereas patients from the private sector reported positively on being a part of the decision-making process. Therefore, patients in the public sector may not be afforded the same opportunity during decision-making as compared with patients in the private sector. This disparity in activities and actions creates a perception of the private sector better adhering to PCC than the public sector. This is supported by Kayyali, Marques Gomes, Mason, and Naik (2016), suggesting the need for improved counselling skills is required to better meet patients’ needs and expectations. Therefore, it can be deduced, improving these skills amongst audiologists may prove to increase patient benefit through shared decision-making, whilst adhering to PCC dimensions.

### Category 4: Perceptions on multicultural sensitivity

Patients seeking audiological services have diverse cultural and linguistic backgrounds that should be considered whilst delivering services (ASHA, 2012). This is substantiated by Owen et al. (2016), reporting that patients who perceived their therapist as less culturally sensitive showed negative outcomes from services. This is further substantiated by Khoza-Shangase and Mophosho (2018), who conducted research focusing on audiology services offered in South Africa, revealing a need to Africanise services to ultimately improve patient benefit and outcomes. However, despite the documented need to Africanise services, all participants in this study described a positive perception of cultural sensitivity. All seven participants described audiologists to be multiculturally sensitive, which is appreciated as South Africa is a multicultural society. The participants attributed the audiologists’ multicultural sensitivity to understanding and respect of their culture as well as the diversity of patients seen in waiting rooms. This study speculates that this positive response demonstrates that audiologists’ undergraduate training on cultural sensitivity is being applied by graduates in their clinical practice. Furthermore, Participants 1 and 2 report on the compassionate bedside manner of the audiologist, which is positive. Participant 2 (45, Female) said, ‘I like that she respected the fact that I was covered and she didn’t show any irritation when I took my time to remove my scarf’.

All participants further attributed their audiologists’ multicultural sensitivity to showing kindness and respect for their customs during practice; in turn, all participants described their experience of counselling to be pleasant, possibly attributed to their understanding of multicultural sensitivity, which this study appreciated. This is substantiated by Owen et al. (2016), who reported that patients who perceived their therapist as less culturally sensitive showed adverse outcomes from services. This was further verified by this study, where the perception of cultural sensitivity by the patient was positive as portrayed in the following narrative. Participant 1 said, ‘I think we were treated with the highest respect and honour and decency and kindness’.

The patient’s positive perceptions of multicultural sensitivity is supported by literature, advocating for multicultural sensitivity in providing holistic management for patients for increased patient benefit (De Man et al., 2016). This is substantiated by De Man et al. (2016), generating a link between patient satisfaction from PCC approaches and multicultural service delivery.

In addition, the participant’s perception of cultural sensitivity differs from that of the audiologist, as issues of their own cultural beliefs were not considered to be part of cultural sensitivity. Cultural beliefs play an integral part in multicultural sensitivity, as supported by Govender and Khan (2017) documenting that patients have cultural beliefs related to the cause of hearing loss and suggesting further training and need for audiologists to demonstrate improved cultural competence, suggesting that audiology training on cultural sensitivity is different from how these patients perceive cultural sensitivity. Another disjuncture between audiologists and patients in terms of cultural sensitivity is identified in this study, as with the initial study. Both studies have found that there is no similar understanding of the definition and application of cultural sensitivity. However, finding a consensus is not the focus of this study. Furthermore, De Man et al. (2016) suggested that the ability of audiologists to include and respect multicultural services aids in promoting patient satisfaction.

### Category 5: Perceptions on patient satisfaction

All participants positively described their overall benefit and satisfaction from counselling practices. Participants attributed this to the skill set of the audiologists, as they were described as kind, patient and understanding. All participants reported to have benefited from counselling and attributed this benefit to their understanding of their hearing impairments as seen in the given narrative. Furthermore, they expressed satisfaction with the services received:

‘It did [*meet expectations*], with my understanding and acceptance of the hearing loss, I’m still a bit scared about it. The counselling session did help me with accepting my hearing loss.’ (Participant 6, 34, Female)

Apart from understanding their hearing loss, all participants reported satisfaction from their counselling as it led to compensating through hearing devices, which has improved their quality of life (Parham et al., 2013). This is laudable as it demonstrated that audiology practice for patients with presbycusis and associated tinnitus is meeting their expectations, which has a positive impact on their outcomes. Moreover, participants reported satisfaction based on the audiologist’s skills as seen in the given narrative.

Participant 2 (45, Female) said, ‘I don’t have any complaints. I felt that I was comfortable. I was at ease, I don’t have any complaints, she was sweet, she was polite, she was patient’.

The given narrative indicates that the patient reported satisfaction because the audiologist’s demeanour was demonstrated during counselling, which is supported by literature noting audiologists with essential skills conducive to counselling will increase patient satisfaction (Alex et al., 2019). This finding is further substantiated by English and Archbold (2014), based on a study in the United Kingdom on a group of 20 audiologists attending a programme to expand their knowledge on counselling. The study concluded that PCC may improve patient–audiologist dynamics and patient satisfaction, which is inherent with findings from this study. The initial study on audiologists’ counselling practices coupled with this study has found that evaluation of patient satisfaction is not routinely performed, which is concerning within the PCC framework as the patient’s perception and satisfaction has not been extensively incorporated.

### Category 6: Recommendations to improve counselling practices

Patient-centred care dimensions focus on the patient’s individual needs and preferences, which are used to drive services to improve patient benefit and satisfaction (Balachandran, 2020). Therefore, heeding patients’ recommendations may lead to improved counselling practices specific to the context of KZN. Participants provided recommendations towards improving counselling practice in the following points. Firstly, participants’ recommendations were to extend the time of sessions to better understand their patients, as can be seen in the following narrative:

‘The counselling session was a little bit pressurised and maybe there were many patients and the time is short and the audiologist it is not sensitive to the particular needs and just wants to rush through things and get the job done, it is in those constraints, they may not necessarily pick up deeper-lying issues it won’t surface unless time is given to hear the person out and they might just jump to conclusions they make on just thinking that they understand the situation and then act accordingly.’ (Participant 1, 67, Male)

Increasing the number of sessions or split sessions may help to avoid overwhelming a patient with volumes of information:

‘I would’ve broken it down, I would seat the patient down and explain what’s wrong firstly and do what my audiologists did and tell them there is no medication to help the problem that I have and then tell the patient that these are your options I can fit you with a hearing aid and see how that goes and things like that and then do it on another day.’ (Participant 2, 45, Female)

Secondly, participants recommend including a significant other in the counselling session as well as an interpreter in case of language differences:

‘I would like when a patient is being seen and there is a language barrier, I would like when that patients is seen thats not speaking English to have an isiZulu speaking translator during the appointment.’ (Participant 5, 69, Female)

Thirdly, participants recommend providing more information on hearing impairment or vocational possibilities for those with a hearing loss that may be shared with others out of the counselling session. Participant 6 (34, Female) said, ‘I think they need to add more advice or information about what jobs we can do with a hearing loss or what jobs we can go into’.

These recommendations by participants provide audiologists with insight into first-hand perceptions of patients to better implement PCC in practice.

## Study limitations and strengths

This study provides a perspective from a group of participants with presbycusis and associated tinnitus from different districts that are inclusive of urban and rural areas with different stratification of the population. Furthermore, the inclusion of private and public sectors provides for different perspectives and lived experiences by the participants. Views of a smaller sample population cannot represent the entire population as the sample was limited in terms of diversity.

## Conclusion

There is limited research on patients’ perceptions of counselling practices in audiology (Meibos et al., 2017), thus creating a knowledge gap that has contributed to the insufficiencies of implementing PCC into audiology counselling practices, inadvertently impacting patient outcomes. This study found positive perceptions where information counselling was dominant over adjustment counselling. There is a need for audiologists to increase focus on adjustment counselling to address the needs of the patients towards adjusting and living with presbycusis and associated tinnitus. There is concern related to patient confidence and ability to relate their diagnosis and assistive device; it was found to be limited, suggesting gaps in informational counselling practice by audiologists. This creates a need for improvement in informational exchange during counselling. Positive perception and preference for individual over group counselling sessions was reported; however, audiologist are urged to discuss the benefit of group sessions and offer the option as part of the service package. Positive perceptions towards tools to assess efficacy were observed, but there are diverse levels of knowledge and use of efficacy tools between audiologists and patients, suggesting desynchrony in the understanding of tools between audiologist and their patients related to tool use during counselling. Therefore, further research is required to explore the efficacy and patient benefit from these tools. Furthermore, as part of their practice, audiologists need to explain the tools and their benefit to patients and conduct verification based on objective tools. There was a positive regard for and confidence in the audiologists’ training and perceived competence, with caution on the disparity between the competency as rated by the audiologist versus the patients; this needs further exploration. Patients considered their audiologists to have adequate training and competence if they demonstrated empathy and shared decision-making. The patient’s perceptions were inclined to show satisfaction and benefit towards counselling practices currently being conducted by audiologists within KZN. These practices when exploring patients’ perceptions suggested that audiologists are implementing PCC dimensions into practice. However, further research can assist in identifying barriers and subsequently evaluate and improve implementation of PCC in the field of audiology holistically. However, this study’s findings on patients’ recommendations on improving counselling closely resembled PCC dimensions and the conceptual framework depicted in this study in terms of implementing the eight Picker principles. These recommendations made by participants further assist in closing the knowlegde gap in practice. However, further research if requried on effective ways of implementing PCC into practice (Meibos wt al., 2017). However, further research is needed to provide quantitative evidence on the efficacy of counselling practices in line with PCC, as this study provided the qualitative aspect.
